# Expression of Dystrophin Dp71 Splice Variants Is Temporally Regulated During Rodent Brain Development

**DOI:** 10.1007/s12035-024-04232-2

**Published:** 2024-05-28

**Authors:** Mayram González-Reyes, Jorge Aragón, Alejandra Sánchez-Trujillo, Griselda Rodríguez-Martínez, Kevin Duarte, Evangelia Eleftheriou, Jean-Vianney Barnier, Delphine Naquin, Claude Thermes, José Romo-Yáñez, Jérome E. Roger, Alvaro Rendon, Cyrille Vaillend, Cecilia Montanez

**Affiliations:** 1 Departamento de Genética y Biología Molecular, Centro de Investigación y de Estudios Avanzados del Instituto Politécnico Nacional (CINVESTAV), Mexico City, Mexico; 2 Institut des Neurosciences Paris Saclay, Université Paris-Saclay, CNRS, Saclay, 91400 France; 3 Institut de la Vision, Sorbonne Université-INSERM-CNRS, 17 rue Moreau, Paris, 75012 France; 4 Present Address: Laboratorio de Investigación en Inmunología y Proteómica, Hospital Infantil de México Federico Gómez, Mexico City, Mexico; 5 Institute for Integrative Biology of the Cell (I2BC), Université Paris-Saclay, CEA, CNRS, Gif-sur-Yvette, 91198 France; 6 Present Address: Coordinación de Endocrinología Ginecológica y Perinatal, Instituto Nacional de Perinatología, Mexico City, Mexico; 7 CERTO-Retina France, Saclay, 91400 France

**Keywords:** Duchenne muscular dystrophy, Dp71 isoforms, Fetal brain, Postnatal brain development, Hippocampus, cortex, cerebellum, Nanopore sequencing

## Abstract

Dystrophin Dp71 is the major product of the Duchenne muscular dystrophy (*DMD*) gene in the brain, and its loss in DMD patients and mouse models leads to cognitive impairments. Dp71 is expressed as a range of proteins generated by alternative splicing of exons 71 to 74 and 78, classified in the main Dp71d and Dp71f groups that contain specific C-terminal ends. However, it is unknown whether each isoform has a specific role in distinct cell types, brain regions, and/or stages of brain development. In the present study, we characterized the expression of Dp71 isoforms during fetal (E10.5, E15.5) and postnatal (P1, P7, P14, P21 and P60) mouse and rat brain development. We finely quantified the expression of several Dp71 transcripts by RT-PCR and cloning assays in samples from whole-brain and distinct brain structures. The following Dp71 transcripts were detected: Dp71d, Dp71d_∆71_, Dp71d_∆74_, Dp71d_∆71,74_, Dp71d_∆71−74_, Dp71f, Dp71f_∆71_, Dp71f_∆74_, Dp71f_∆71,74_, and Dp71f_Δ71−74_. We found that the Dp71f isoform is the main transcript expressed at E10.5 (> 80%), while its expression is then progressively reduced and replaced by the expression of isoforms of the Dp71d group from E15.5 to postnatal and adult ages. This major finding was confirmed by third-generation nanopore sequencing. In addition, we found that the level of expression of specific Dp71 isoforms varies as a function of postnatal stages and brain structure. Our results suggest that Dp71 isoforms have different and complementary roles during embryonic and postnatal brain development, likely taking part in a variety of maturation processes in distinct cell types.

**Supplementary Information:**

The online version contains supplementary material available at 10.1007/s12035-024-04232-2.

## Introduction

The Duchenne muscular dystrophy (DMD) syndrome is caused by mutations within the large *DMD* gene located on the X chromosome (Xp21). A variety of cognitive and behavioral dysfunctions observed in both DMD patients and mouse models has been attributed to the loss of distinct brain dystrophin proteins normally expressed in the brain by this multi-promoter gene. However, the localization of brain dystrophins and their putative role during brain development and function is still largely unclear. The *DMD* gene has seven promoters, three of which express full-length dystrophin proteins of 427 kDa (Dp427) in the brain (Dp427b), muscle (Dp427m), and cerebellar Purkinje cells (Dp427p), while downstream promoters are responsible for the expression of shorter C-terminal dystrophins expressed in the nervous system (Dp260, Dp140, Dp116, and Dp71) (reviewed in [1, 2]). Among those, Dp71 is generating a great deal of interest in understanding DMD neuropathology. Indeed, Dp71 is the main product of the *DMD* gene in the central nervous system (CNS), and its deficiency has been associated with the most severe central comorbidities in DMD [3–9].

Dp71 has a general promoter [10] that also encodes a Dp71 isoform truncated at the C-terminus, Dp40 [11–13]. Dp71 expression results in a 4.5-kb transcript that undergoes a variety of alternative splicing events affecting exons 71 to 74, 78 and intron 77, giving rise to three different groups of Dp71 isoforms with specific C-terminal ends due to a frameshift induced by splicing of exon 78 and/or intron 77. The Dp71d group includes Dp71 isoforms containing exon 78, the Dp71f group isoforms lack exon 78, and the Dp71e group contains a part of intron 77 [14–16], yet only the Dp71d and Dp71f groups have been detected in the retina and brain [17]. The alternative splicing of exons 71 to 74 does not change the open reading frame but explains the presence of several Dp71 isoforms, including the full-length Dp71d, the exon 78-spliced Dp71f, and isoforms containing additional patterns of exon 71–74 splicing, such as Dp71d_∆71_, Dp71d_∆74_, Dp71d_∆71,74_, Dp71d_∆71−74_ (Dp71d isoforms), Dp71f_∆71_, Dp71f_∆74_, Dp71f_∆71,74_, and Dp71f_Δ71−74_ (Dp71f isoforms). These Dp71 isoforms are named according to their group and the alternative splicing of exons 71 to 74 as in previously established nomenclature [17].

DMD is associated with variable neurodevelopmental comorbidities including intellectual disability, neuropsychiatric disturbances, and abnormal retinal physiology [18, 19], which are drastically aggravated when mutations impede the expression of Dp71 isoforms [20–24]. Neuropsychological studies in DMD patients and preclinical studies in mouse models suggested that cognitive impairments could be attributed to a dysfunction of the cortico-cerebellar network [25–28], and/or hippocampal-prefrontal cortex network [2, 29, 30], particularly in case of Dp71 deficiency [7, 31]. Abnormalities in brain cell morphology have been reported in both DMD patients and DMD mouse models lacking Dp71, which may include gliosis, dendritic and synaptic abnormalities, and vascular defects [7, 32, 33]. Because brain dystrophins, and particularly Dp71, show variable expression levels from fetal to adult stages, it is believed that they may play different roles in specific cell types, brain regions, and steps during embryonic and postnatal brain development [34–36].

The Dp71 promoter presents a stage- and cell-type specific activity during the development and differentiation of various organs and tissues, which appears to be related to morphogenic events and terminal differentiation [5]. The expression of Dp71 full-length isoform (Dp71d) gradually increases from fetal to adult stages in the CNS [37]. However, we have previously shown that there is a differential expression of Dp71 isoforms in the adult mouse brain and retina [17], raising the hypothesis that different Dp71 isoforms might also show differences in their relative expression at specific stages of CNS development. This variety of isoforms may be important regarding the multiple functions of Dp71, which appears to be involved in the cell cycle, neuronal differentiation, adipose tissue differentiation, nuclear and membrane architecture, synaptic function, and brain/retinal homeostasis through anchoring potassium (Kir4.1) and water (AQP4) channels in glial cell endfeet [8, 9]. This is further supported by our studies in cellular in vitro models showing that distinct Dp71 isoforms have different subcellular localization and colocalization with components of the dystrophin-associated protein complex [7, 38, 39] and may have distinct roles during proliferation and differentiation processes [40–43].

In the present study, we characterized the expression of Dp71 isoform mRNAs from embryonic (E10.5, E15.5) to adult (P60) mouse and rat brain, as well as during the postnatal development (P1, P7, P14, and P21). We also compared distinct brain structures, including mouse hippocampus, cortex, and cerebellum that represent three target brain structures involved in the cognitive deficits reported in DMD patients and/or DMD mouse models. We found expression of Dp71d (GenBank: JN900253), Dp71d_∆71_ (GenBank: KJ480729), Dp71d_∆71−74_ (GenBank: KX525239), Dp71d_∆74_ (GenBank: KX525241), Dp71d_∆71,74_ (GenBank: KX525240), Dp71f (GenBank: KJ480730), Dp71f_∆71_ (GenBank: KJ480731), Dp71f_∆71−74_ (GenBank: KX525238), Dp71f_∆74_ (GenBank: KX525242), previously reported [17], and Dp71f_Δ71,74_ (GenBank: OR911957). Dp71f isoform was the main Dp71 isoform expressed at E10.5, but it showed gradual down-regulation from E15.5 to adult stages, while expression of Dp71d transcripts increased (Dp71d, Dp71d_∆71_, and Dp71d_∆71−74_). Moreover, specific isoforms showed variations in their relative expression during postnatal development in distinct brain structures.

## Materials and Methods

### Animals and Tissue Extractions

Mice of the C57BL/6J strain (Janvier Labs, France) were handled according to guidelines of the Paris-Saclay Institute of Neuroscience in France (agreement D91-471-104) in compliance with European Directive 2010/63/EU and French National Committee (87/848). Following euthanasia of pregnant females at 10 and 15 post-coitum days, embryos (E10.5 and E15.5) were extracted from the uterine cavity, delicately isolated, and deposited in cold phosphate-buffered saline. Embryonic brains were dissected out and placed in 1 ml of cold Trizol Reagent (Invitrogen, France). The hippocampus, cerebral cortex, and cerebellum tissues were dissected out from a mouse at postnatal days P1, P7, P14, P21, and P60. A whole brain was collected from an adult mouse at P60 for comparison with embryonic brains. These mice were killed by cervical dislocation, and tissues were placed in 1 ml of cold Trizol Reagent per 50–100 mg of tissue. For cloning assays at E10.5 and E15.5, one embryo was used at each age (*n* = 1) and three adult mice for P60 whole brain (*n* = 3). For postnatal ages, we compared expression in the hippocampus at P1, P14, and P21 (*n* = 1 per age) and at P7 and P60 (*n* = 2 per age); in the cortex at P21 (*n* = 1) and at P1, P7, P14, and P60 (*n* = 2 per age); and in the cerebellum at P7 and P21 (*n* = 1 per age) and at P1, P14, and P60 (*n* = 2 per age). For nanopore analyses, E10.5 whole brain (*n* = 3) was compared to the cerebellum and hippocampus at P60 (*n* = 2). For RT-PCR assays, the cerebellum, cortex, and hippocampus at P60 were analyzed in two independent experiments (*n* = 2).

Wistar rats were handled according to the regulations approved by CINVESTAV-UPEAL (Unit for Production and Experimentation of Laboratory Animals) and Mexican Official Norm (NOM-062-ZOO-1999). The forebrain, midbrain, and hindbrain were dissected out at E14.5, E16.5, E18.5, E20.5, P1, P4, P7, P14, and P60 and processed as described above. For embryonic stages, a pool of all embryos (5–10) of one pregnant rat was obtained and considered one experiment. For early postnatal stages, a pool of three rat babies was obtained and considered one experiment, and for P60, one rat was used for each experiment. Three independent experiments were carried out for each stage.

### RNA Extraction and RT-PCR Assays

Total RNA was obtained following the Trizol Reagent’s protocol (Invitrogen, France). The quality of RNA and concentration were measured by Nanodrop followed by migration in 1.5% agarose gels prestained with ethidium bromide. The 260/280 ratios of the samples used in this study were between 1.8 and 2, indicating the high quality of RNA, and concentrations ranged from 600 to 4000 ng/µl. RNA integrity verified before using samples for nanopore sequencing indicated RIN > 8.5 (Agilent 2100 Bioanalyzer). Up to 1 µg of total RNA was primed with 200 ng of dystrophin-specific primer (dcDNA, 5′ GAATATTATAAAAACCATGCG) and 1 µl of Oligo dT for reverse transcription using the Super Script III First-Strand Synthesis kit (Invitrogen, France). Briefly, RNA, primers, and dNTPs were incubated at 65 °C for 5 min and placed on ice for 2 min. Then, the cDNA synthesis mix (RT buffer, MgCl_2_, DTT, RNaseOUT, and SuperScript III RT) was added and incubated at 55 °C for 1 h and 85 °C for 5 min. Then, cDNAs were treated with 1 µl of RNase H at 37 °C for 20 min. The Dp71 cDNA (200 ng) from mouse or rat was amplified with AccuPrime™ *Pfx* DNA Polymerase (Invitrogen) using a forward primer directed against the specific 5′UTR of Dp71 (5′ AGTGCTTTCGGCTGCGAGC) and a reverse primer against the last Dp71 exon, mEx79R (5′ TTATTCTGCTCCTTCTTCATCTGTCATGACTG). The PCR reactions were carried out as follows: an initial incubation at 95 °C for 2 min; 35 cycles composed of 95 °C for 30 s and 68 °C for 3 min; and a final extension at 72 °C for 7 min. Dp71 PCR products from rat tissues were re-amplified using the mEx77F (5′ CCTTCCCTAGTTCAAGAG) and mEx79R primers to evaluate the alternative splicing of exon 78 following multiplex PCR conditions (see below). Beta-actin mRNA amplification was used to test the quality of cDNAs (data not shown). The PCR products were visualized in 1.5% agarose gels prestained with ethidium bromide.

### Identification of Dp71 Isoforms by Cloning

**Fig. 1:**
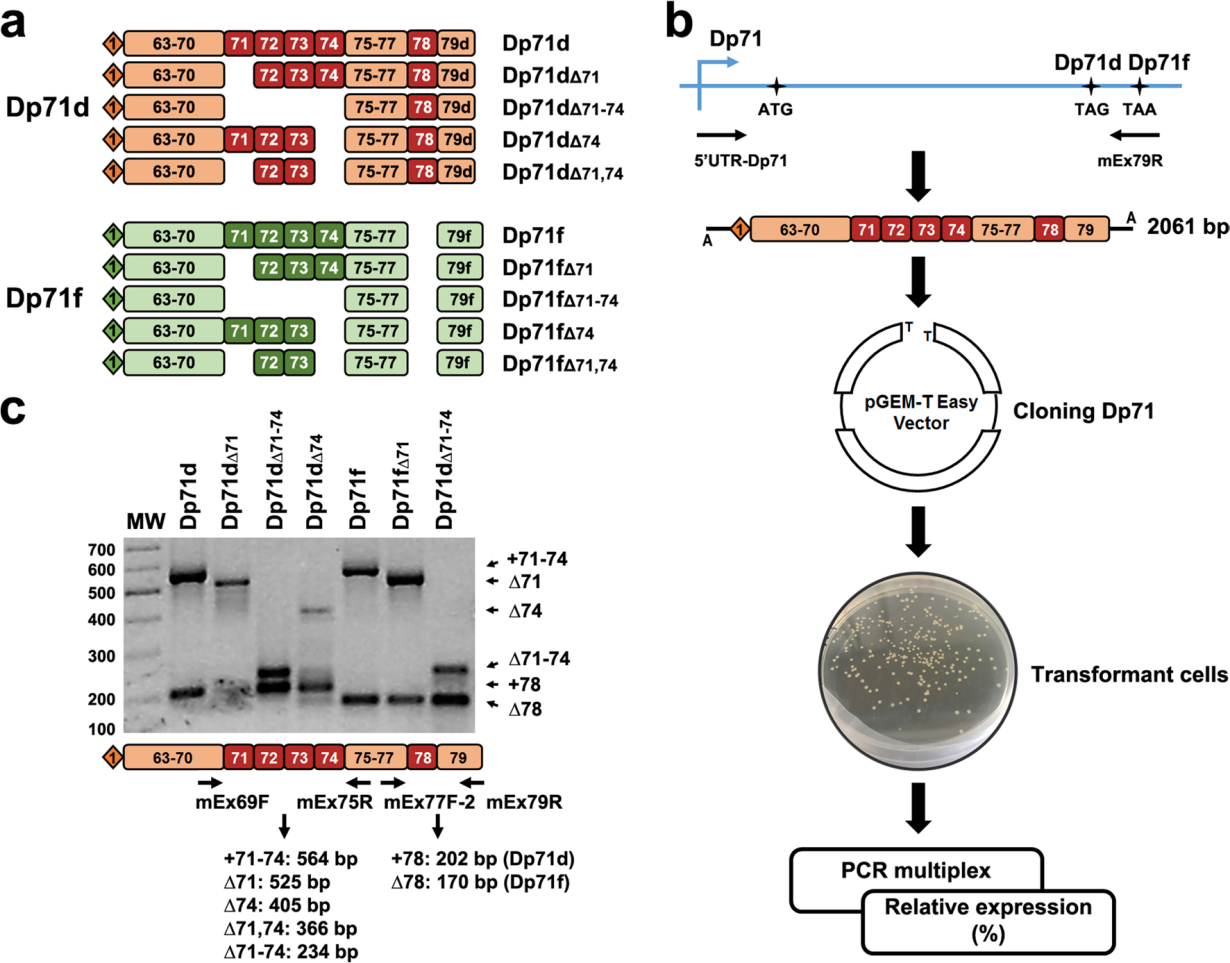
Identification of the Dp71 isoforms. **a** Schemes showing exon composition of Dp71d and Dp71f groups for the Dp71 isoforms found in this work. Exon 79 is labelled 79d and 79f because its reading frame is changed by the skipping of exon 78 in the Dp71f group. **b** Flowchart for the RT-PCR and cloning strategy of the Dp71 isoform expressions. The stop codon for Dp71d and Dp71f groups is indicated (TAG, TAA). Horizontal arrows in the top drawing represent the primers 5′UTR-Dp71 and mEx79R to amplify all Dp71 transcripts (up to 2061 bp for full-length), following the cloning into the pGEM-T-Easy vector and the analyses of *Escherichia coli* transformant cells by multiplex PCR. **c** Electrophoresis of the Dp71 isoform amplification by multiplex PCR. Scheme of the multiplex PCR analyses is shown at the bottom. Arrows represent the primers used to characterize splicing of exons 71 to 74 (mEx69F, mEx75R) and those enabling detection of exon 78 splicing (mEx77F-2, mEx79R). The expected size of PCR products is indicated below the multiplex PCR scheme. MW, molecular weight in base pairs indicated on the left

The Dp71 PCR products were purified using the Nucleo-Spin Gel Clean Up (Macherey Nagel, France) according to the kit protocol. The purified Dp71 products for each tissue were cloned into the pGEM-T Easy vector (Promega, France) and used to transform *Escherichia coli* DH5α cells. Transformant colonies were screened for each tissue to select positive colonies for the Dp71 insert. These transformed cells were examined by multiplex PCR with 20 ng of mEx69F (5′ CATGGTAGAGTATTGCACTCCG) and mEx75R primers (5′ GGAGGAGATGGCAGTGGAGAC) to characterize the splicing of exons 71 to 74 and with 30 ng of mEx77F-2 (5′ CTCCCCAGGACACAAGCACAG) and mEx79R primers to characterize the splicing of exon 78, in a final volume of 25 µl. Multiplex PCR was run with an initial incubation at 95 °C for 10 min; 40 cycles composed of 95 °C for 30 s, 55 °C for 30 s, and 72 °C for 30 s; and a final extension at 72 °C for 7 min. PCR products were visualized in 1.5% agarose gels prestained with ethidium bromide. The relative expression (%) for each Dp71 isoform was obtained by counting the colonies positive for each Dp71 isoform from at least a hundred Dp71-positive colonies for most samples in cloning experiments. At least one of each identified Dp71 isoform was analyzed by plasmid DNA sequencing using the Dye Deoxy Terminator Cycle Sequence Kit (Applied Biosystems, Foster City, CA, USA) and aligned to the annotated sequence of Dp71 using in silico analyses. Figure 1a shows all the Dp71 isoforms identified in this work.

### Identification of Dp71 Isoforms by Nanopore Sequencing

The cDNA from a selection of samples used for the cloning strategy was also used for the construction of Oxford Nanopore Technology (ONT) DNA libraries. The PCR products were treated with 1 µl of RNAse H for 20 min at 37 °C, amplified as described above, visualized in 1.5% agarose gels revealing a large band of the expected size (1.7–2 kb), and then purified using the BioBasic EZ-10 spin column DNA Miniprep Kit (Gentaur France SARL). Libraries were then constructed following the native barcoding genomic DNA protocol (version NBE_9065v109_revL_14Aug2019) using the SQK-LSK109 kit in combination with the EXP-NBD104 kit. Libraries were sequenced using R9.4.1 flow cells on the ONT GridION system. The sequencing run yielded between 0.76 and 1.355 M reads per sample on GridION (release 20.06.09). Base calling was performed using the Oxford Nanopore base caller Guppy (v4.0.11). Reads (minimum quality mean 7) were demultiplexed and trimmed with Guppy. Dp71 transcripts harbor 18 exons that may differentially contribute to annotated isoforms that we identified and quantified using FLAIR (v1.4.0) [44]. For each sample, the relative expression of an isoform was computed by dividing the number of reads corresponding to this isoform divided by the total number of analyzed reads. Among all isoforms identified by Flair, only those with a relative expression larger than 0.1% were further considered.

### Statistical Analyses

The quantitative data are presented as the mean percent of colonies positive for each Dp71 isoform or group of isoforms (Dp71d and Dp71f), from at least a hundred Dp71-positive colonies per animal for most samples in cloning experiments, or as the percent of reads corresponding to each Dp71 isoform or group of isoforms with relative expression > 0.1% (nanopore sequencing). The mean percent was calculated when two or three independent experiments (*n* = 2–3 mouse samples) were performed. The proportions of Dp71 isoforms were compared across ages and/or among brain structures using chi-square analyses of contingency tables, to evaluate relationships between these variables and the variation in the relative expression of Dp71 isoforms, and for comparisons with theoretical homogenous/random distribution across ages and structures. Variations were considered significant when *p* values were < 0.01 (StatView 4.57, Abacus Concepts, Inc., Berkeley, CA, USA).

## Results

### Dp71 Isoforms in Embryonic and Adult Rodent Brains

In a previous work, we have shown that adult mouse brain and retina express several Dp71 transcripts grouped as the Dp71d and Dp71f groups of isoforms, as illustrated in Fig. 1a [17]. Here, we followed the same strategy to characterize the relative expression of Dp71 isoforms at different stages of brain development, from embryonic to postnatal and adult ages. The Dp71 mRNA was first reversed transcribed and amplified using primers corresponding to the specific 5′UTR of Dp71 and its last exon (exon 79 of the DMD gene), thus enabling amplification of most brain Dp71 isoforms described to date [17], except the short N-terminal isoform called Dp40 that could not be amplified with this method. The Dp71 PCR products were then purified and cloned into the pGEM-T Easy vector that was used to transform *Escherichia coli* DH5α cells (Fig. 1b). The positive colonies for Dp71 were characterized by multiplex PCR using the following primer pairs: mEx69F and mEx75R, mEx77F-2 and mEx79R. This methodology allowed the identification of the alternative splicing of exons 71 to 74 and exon 78 respectively, as previously described [17]. A typical result of multiplex PCR for these Dp71 isoforms is shown in Fig. 1c. For each Dp71 isoform, the upper bands correspond to the expected size of the region encompassing exons 71 to 74, including or not the different alternative splicing that can affect these exons. The lower bands correspond to different splice variants containing or not the exon 78. Isoforms of the Dp71e group containing a part of intron 77 were not detected in our samples.

**Fig. 2:**
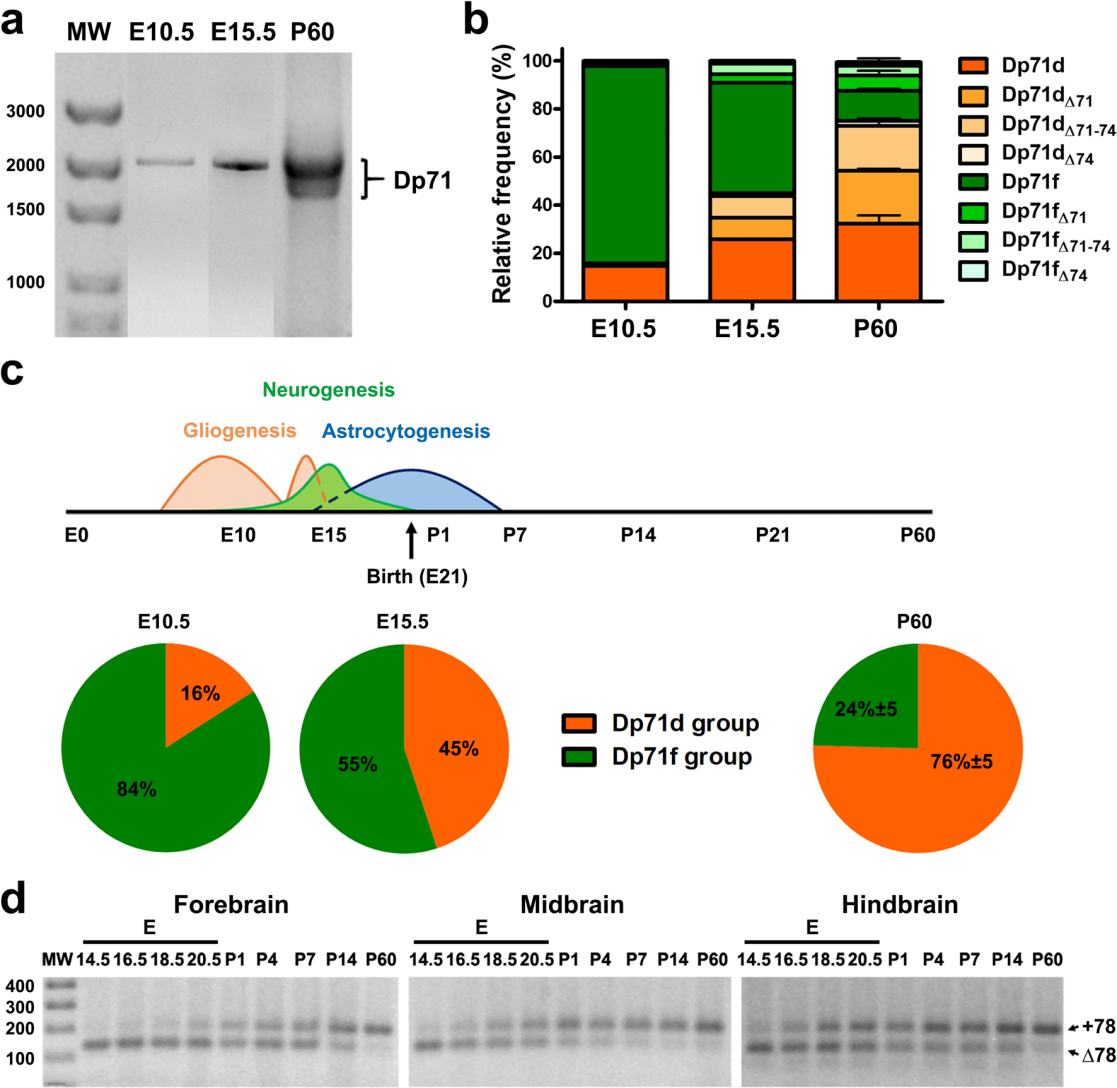
Comparison of the expression of Dp71 isoforms in embryonic and adult mouse (**a**–**c**) and rat (**d**) brain. **a** Electrophoresis showing amplification of Dp71 isoforms from total embryonic (E10.5 and E15.5) and adult (P60) mouse brain using primers corresponding to the specific 5′UTR of Dp71 and its last exon (exon 79). **b** Plots showing the relative expression of Dp71 isoforms in mouse embryonic brain at E10.5, E15.5, and P60. **c** Graphs show the relative expression (%) of Dp71d (orange) and Dp71f (green) isoforms in embryonic (E10.5 and E15.5) and adult (P60) brain. Gliogenesis (that includes radial glial cells and microglia invasion), neurogenesis, and astrocytogenesis are indicated at the different development stages of mouse brain. **d** Electrophoresis showing the presence or absence of exon 78 (+ 78, Δ78) in Dp71 transcripts of embryonic (E14.5 to E20.5), postnatal (P1 to P14), and adult (P60) rat forebrain, midbrain, and hindbrain (as indicated). Data are shown as percent or mean percent of the relative frequency plus SD (bars or values). MW, molecular weight in base pairs is indicated on the left of figures **a** and **d**

To characterize the regulated expression levels of distinct Dp71 isoforms during mouse brain development, we first analyzed their relative expression in whole-brain samples at E10.5 and E15.5 embryonic stages and in the adult at P60. The whole-brain samples were analyzed by RT-PCR (Fig. 2a) and cloning assays to obtain the relative expression of each Dp71 isoform, expressed as the percent of total Dp71 mRNAs. As shown in Fig. 2b, the cloning analyses revealed that the E10.5 brain mainly expresses the Dp71f isoform (82%) and then Dp71d (15%), while at E15.5, the expression level of Dp71f was decreased to 46% and Dp71d was found at 26%. Other Dp71 isoforms of each group were detected at lower levels in these samples. In marked contrast, the adult mouse brain comprised 32% of Dp71d, 22% of Dp71d_Δ71_, 19% of Dp71d_Δ71−74_, and 13% of Dp71f (Fig. 2b). Putative over-sampling of the 3′ end of Dp71 transcripts might be expected from this method, due to early fall-off of RT enzymes, easier amplification, and/or preferred incorporation of the smaller amplicons during cloning. However, it is noteworthy here that the shorter Dp71 isoforms were usually less represented, or absent (e.g., at E10.5), than the longer or full-length isoforms and showed important variations as a function of the developmental stage. Moreover, most results were confirmed by RT-PCR or nanopore sequencing methods (described below), which does not confirm a risk for a quantitative bias in favor of shorter splice variant in the present study.

Hence, the Dp71f, Dp71d, Dp71d_Δ71_, and Dp71d_Δ71−74_ isoforms appear to be differentially regulated from embryonic stage to adulthood. The data are summarized by a group of isoforms (Dp71d and Dp71f) in Fig. 2c. Dp71f was the main Dp71 group of isoforms expressed in the fetal brain, representing 84% at E10.5 (Dp71d vs. Dp71f proportions, *p* < 0.0001; chi-square analysis) that corresponds to the first peak of gliogenesis (radial glial cells and microglia). At E15.5, which corresponds to the peak of neurogenesis and start of astrogenesis, the Dp71f group was partly replaced by isoforms of the Dp71d group and only represented 55% of the detected isoforms (Dp71d vs. Dp71f proportions, *p* > 0.5). Finally, the Dp71f group showed only 24% expression in the adult brain that mainly contained isoforms of the Dp71d group (76%) (Dp71d vs. Dp71f proportion, *p* < 0.0001). The variations in the relative distribution of these two groups of isoforms were significantly different between ages (E10.5 vs. E15.5, *p* < 0.0001; E10.5 or E15.5 vs. P60, *p* < 0.0001). Overall, this shows that the fetal brain mainly expresses Dp71 isoforms lacking exon 78 (Dp71f group), while the adult brain mainly expressed Dp71 isoforms that contain exon 78 (Dp71d group).

To evaluate if the regulation of the Dp71f and Dp71d groups of isoforms between the embryonic and adult stages could be generalized to the rodent brain, we also studied the expression of these two groups of Dp71 isoforms containing or not exon 78 in rat brain samples. In this experiment, the rat forebrain, midbrain, and hindbrain were dissected out at E14.5, E16.5, E18.5, and E20.5 and at postnatal days P1, P4, P7, P14, and P60, and were then processed for RT-PCR assays. Amplified Dp71 transcripts were re-amplified using the mEx77F (5′CCTTCCCTAGTTCAAGAG) and mEx79R primers to evaluate the alternative splicing of exon 78 in Dp71 isoforms (Fig. 2d). Strikingly, we found that isoforms with a spliced exon 78 (Dp71f group) were predominant in the embryonic stages for the three analyzed tissues, forebrain, midbrain, and hindbrain, detected at high levels at E14.5, then gradually decreasing during development until adulthood. Conversely, the isoforms containing exon 78 (Dp71d group) were detected at very low levels at E14.5, but their expression gradually increased during development, reaching high levels at P60, thus mirroring the regulation of Dp71f isoforms as demonstrated in the mouse brain.

### Dp71 Isoforms During Postnatal Development of Mouse Brain Structures

**Fig. 3:**
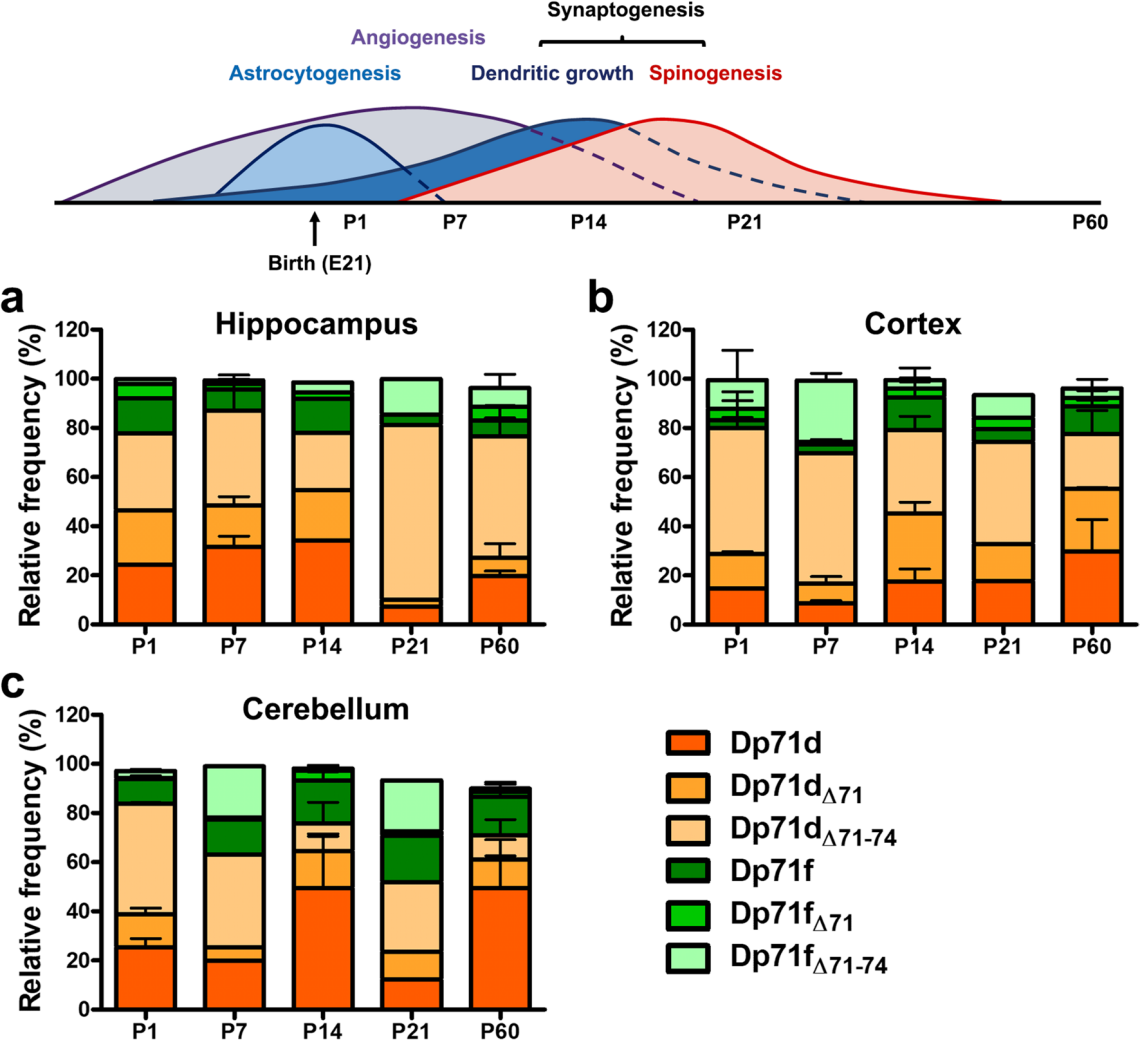
Relative expression of the Dp71 isoforms during the postnatal development of mouse hippocampus, cortex, and cerebellum. Relative expression of the most frequent Dp71d (orange color codes) and Dp71f (green color codes) isoforms (rounded % of total colonies expressing these isoforms) during the postnatal (P1, P7, P14, P21, and P60) development of hippocampus (**a**), cortex (**b**), and cerebellum (**c**). Data are shown as percent or mean percent of the relative frequency plus SD. Astrocytogenesis, angiogenesis, and synaptogenesis (dendritic growth and spinogenesis) are indicated at the different postnatal stages of mouse brain

Dystrophin Dp71 is mostly expressed in non-muscle tissues, and its expression gradually increases during CNS development from the embryonic stage to adulthood [5, 37, 45]. To address the expression of Dp71 isoforms during the postnatal development of mouse hippocampus, cortex, and cerebellum, we analyzed Dp71 mRNAs extracted from these structures at different postnatal days, i.e., P1, P7, P14, P21, and P60. These time points have been selected as they represent gross developmental landmarks of peak astrocytogenesis (P1), angiogenesis (P7), and synaptogenesis, including dendritic growth (P14) and spinogenesis (P14-P21) [46–52]. These developmental landmarks are depicted in the top diagram of Fig. 3. This diagram enables a rapid overview of the main waves of cellular changes taking place during brain development, yet their precise dynamics may substantially differ between cortical, subcortical, and cerebellar structures. The relative expression for each Dp71 isoform was determined by RT-PCR and cloning assays as above. We identified the expression of several main Dp71 isoforms, namely Dp71d, Dp71d_Δ71_, and Dp71d_Δ71−74_, and low levels of Dp71f, Dp71f_Δ71_, and Dp71f_Δ71−74_ (Fig. 3; Supplementary Figure S1). The Dp71d_Δ74_, Dp71d_Δ71,74_, Dp71f_Δ74_, and Dp71f_Δ71,74_ isoforms were also detected in a very low proportion of colonies (< 5%) or absent depending on the tissue and developmental stage (Supplementary Figure S2).

We further analyzed the variation in the expression of six isoforms (Dp71d, Dp71d_Δ71_, Dp71d_Δ71−74_, Dp71f, Dp71f_Δ71_, and Dp71f_Δ71−74_) showing major expression across postnatal ages (P1, P7, P14, P21, and P60), as shown in Fig. 3. The relative expression of these isoforms belonging to each group (Dp71d, Dp71f) significantly varied across postnatal ages in the three brain structures (*p* < 0.0001, for each group of isoforms in each brain structure). Moreover, at each age, the proportion of these isoforms was significantly different from a random equivalent distribution (i.e., different from 17% for each isoform). Overall, we found that the hippocampus, cortex, and cerebellum mainly express Dp71d isoforms throughout the postnatal development. Interestingly, Dp71d_Δ71−74_ showed the higher expression in the hippocampus with a marked peak at P21 (71%), and still relatively high expression at P60 (49%), while in the cortex and cerebellum, this isoform was more abundant (38–53%) at P1 and P7 (Fig. 3a–c). In the hippocampus, the expression of Dp71d and Dp71d_Δ71_ was substantial from P1 to P14 (17 to 34%), but this was then reduced below 10% at P21. Dp71d was the main isoform found in the cerebellum at P14 and P60 (49%), while Dp71d_Δ71−74_ showed maximal expression at P1 (45%) and P7 (38%) in this structure and then decreased in the next postnatal stages. The other isoforms from the Dp71d and Dp71f groups, even if they were expressed at lower levels, also showed variations in their relative level of expression depending on the postnatal stage. We also analyzed the expression of Dp71 isoforms lacking exons 71 to 74 in the adult brain tissues (P60) using standard RT-PCR, and we compared their expression to that obtained in the cloning experiments (Supplementary Figure S3). RT-PCR could not discriminate whether these isoforms belonged to the Dp71d or Dp71f groups, and we, therefore, quantified a band that likely corresponded to Dp71d_Δ71−74_ plus Dp71f_Δ71−74_ expression. This was also compared to the summed relative expression of these two isoforms in the cloning experiment. Both methods were in line to confirm that Dp71 isoforms lacking exons 71–74 are more largely expressed in the hippocampus (> 50%), then in cortex (~ 25–28%), and cerebellum (10–12%). In all, our results thus confirm that the Dp71d group of isoforms is mainly expressed during postnatal stages, as suggested by our initial comparison of embryonic and adult whole-brain samples. This more detailed analysis from P1 to P60 further shows that the expression of most Dp71 isoforms is differentially regulated across postnatal ages.

**Fig. 4:**
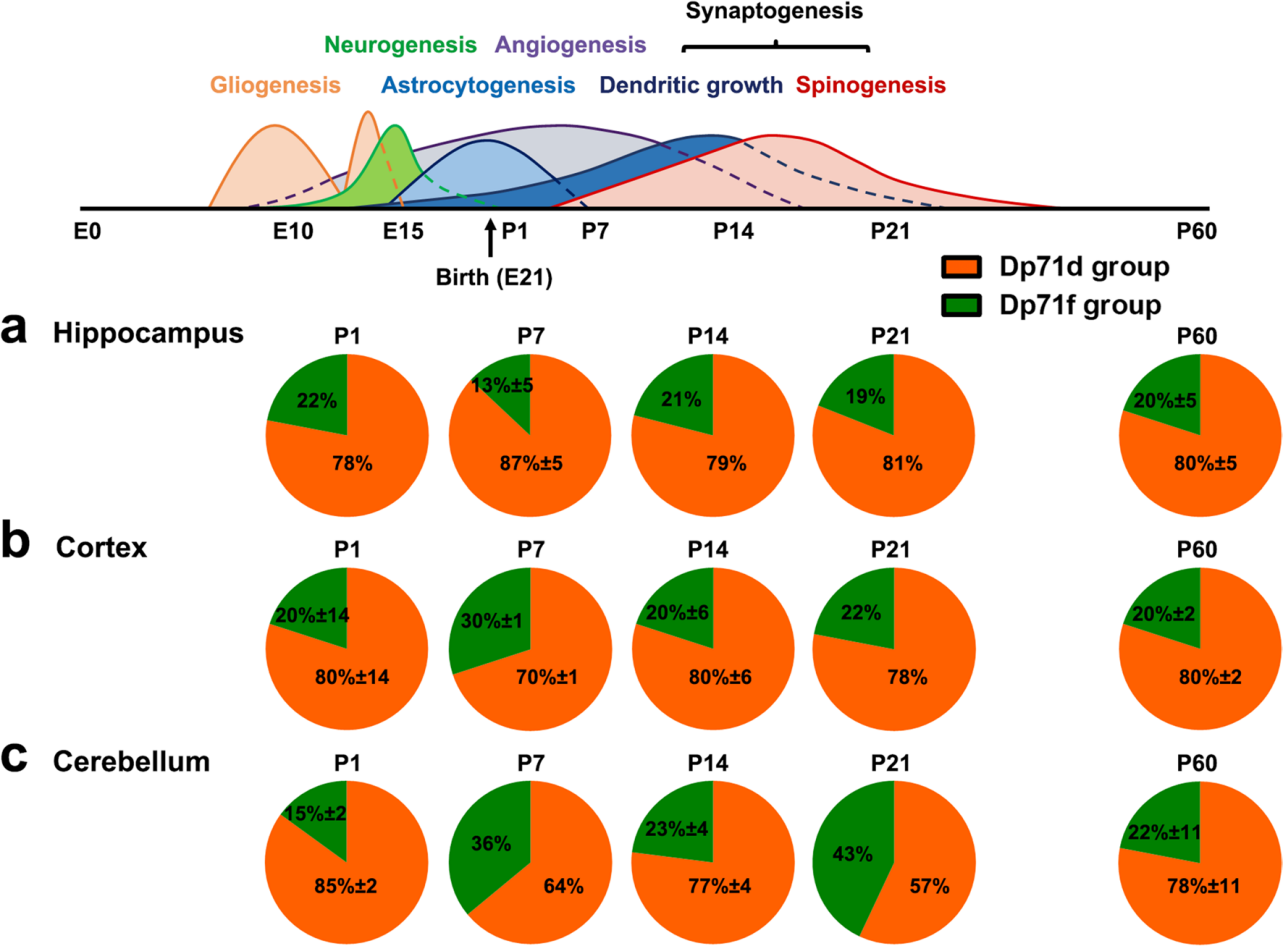
Comparison of the expression of Dp71d and Dp71f isoforms during the postnatal development of mouse hippocampus, cortex, and cerebellum. Expression of Dp71 isoforms during the postnatal (P1, P7, P14, P21, and P60) development of the hippocampus (**a**), cortex (**b**), and cerebellum (**c**). Graphs show the relative expression (%) of Dp71d (orange) and Dp71f (green) isoforms. Data are shown as percent or mean percent of the relative frequency plus SD. Gliogenesis, neurogenesis, astrocytogenesis, angiogenesis, and synaptogenesis are indicated at the different development stages of mouse brain

Figure 4 presents the variable expression of the main groups of isoforms, the Dp71d and Dp71f groups, during postnatal stages. In the hippocampus, the Dp71d group was predominant at all postnatal stages (≥ 78%), apart from slight variations across stages (78 to 87% depending on the stage). In the cortex and cerebellum, we noticed a variation at P7, which globally corresponds to a period of intense angiogenesis and synaptogenesis, as the proportion of Dp71d group of isoforms decreased to 70% and 64%, respectively. In both cases, the increased representation of the Dp71f group was specifically associated with an increase in the expression of the Dp71f_Δ71−74_ isoform between P1 and P7, from 12 to 25% in cortex and 3 to 21% in cerebellum (as shown in Fig. 3). Notably, there was also a large decrease in expression of the Dp71d group in the cerebellum at P21 (57%), which globally corresponds to the end of the synaptogenesis period. This was largely due to the decreased expression of the Dp71d isoform at P21 (12%) compared to P14 (49%), as shown in Fig. 3.

### Nanopore Sequencing as an Alternative Tool for the Detection of Dp71 Isoforms

**Fig. 5:**
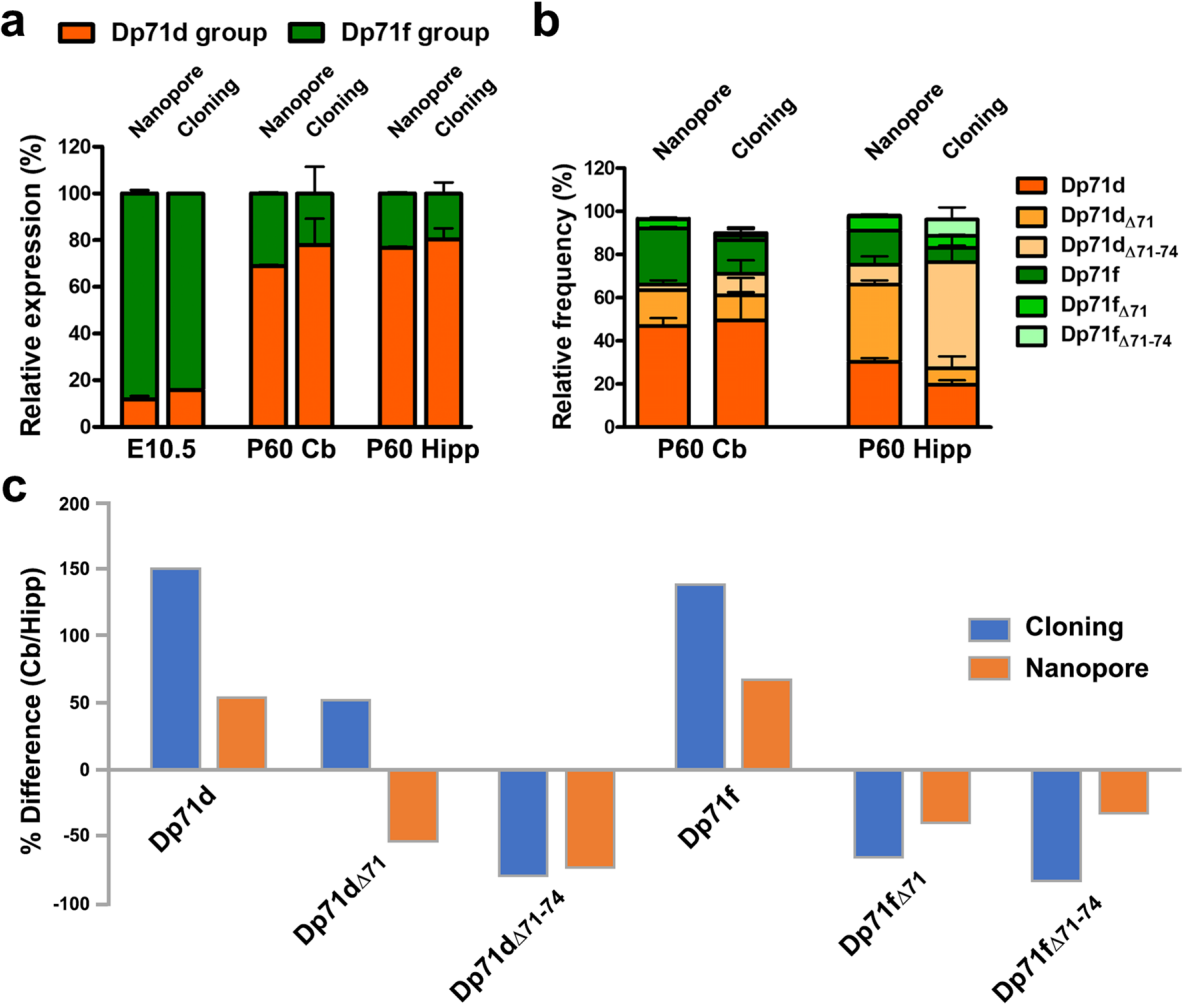
Comparison of Dp71 isoform expression in the mouse brain by nanopore sequencing and cloning. **a** Relative expression (%) of the Dp71d (orange) and Dp71f (green) groups of isoforms at embryonic day 10 (E10.5) and in adult (P60) cerebellum (Cb) and hippocampus (Hipp). **b** Relative expression (%) of the main Dp71 isoforms in adult mouse (P60) cerebellum and hippocampus quantified by nanopore sequencing and cloning experiments. **c** Mean relative difference (% difference) in expression of Dp71 isoforms (as indicated along the X-axis) in adult (P60) cerebellum and hippocampus, calculated as a ratio: (% expression in cerebellum/% expression in hippocampus × 100) − 100. Positive ratio indicates larger expression in cerebellum; negative ratio indicates larger expression in hippocampus. The percent difference is shown from data obtained in the cloning experiments (blue bars) or by nanopore sequencing (orange bars). Data are shown as percent or mean percent of the relative frequency plus SD (**a** and **b**)

In order to evaluate these variations in Dp71 isoform expression with another method, we assessed the quantification of a selection of brain samples using nanopore sequencing. Third-generation sequencing technologies have undergone active development and may find interesting applications in the near future regarding the rapid identification and quantification of splice variants within tissue samples. We, therefore, explored the potential of such strategies to analyze the expression levels of Dp71 isoforms. First, we compared the relative expression of Dp71 isoforms in the embryonic brain (E10.5) and in adult (P60) tissues prepared from cerebellar and hippocampal samples. Ten Dp71 isoforms with a relative expression larger than 0.1% were detected with this method (Dp71d, Dp71d_Δ71_, Dp71d_Δ71−74_, Dp71d_Δ71,74_, Dp71d_Δ74,_ Dp71f, Dp71f_Δ71_, Dp71f_Δ71−74_, Dp71f_Δ71,74_, Dp71f_Δ74_), which corresponds to the same isoforms detected by the cloning method above, except for Dp71d_Δ71,74_ that was not detected in the cloning experiments. In the embryo, Dp71f and Dp71d isoforms showed larger expression compared to the other isoforms (< 1.5%), and the levels quantified by nanopore sequencing and cloning method were close to each other (Dp71f, 87% vs. 82%, respectively; Dp71d, 11% vs. 15%, respectively). We then pulled the data of the different isoforms in their respective groups (Dp71d and Dp71f groups) for comparative analysis of fetal and adult samples. As shown in Fig. 5a, the relative expression of the two groups of isoforms significantly differed between E10.5 and P60 samples (*p* < 0.0001) and was different from the chance level (50%) at each time point (*p* < 0.01). This fully confirmed the result obtained with the cloning strategy, which also demonstrated that the Dp71f group predominates at the E10.5 embryonic stage, while the Dp71d group predominates in adult brain tissues (nanopore, cerebellum 69% and hippocampus 77%; cloning, cerebellum 78% and hippocampus 80%).

In the adult cerebellar and hippocampal samples, however, the relative levels of expression of Dp71 isoforms were different if we compare nanopore sequencing and cloning methods. This was not due to major variability between the two independent experiments used for the nanopore sequencing, which showed homogeneous results. The difference between the two methods was largely influenced by the level of Dp71d_Δ71−74_ which was much lower according to nanopore sequencing compared to the cloning method (Fig. 5b). This suggests that the expression of this isoform was either overestimated by the cloning method or underestimated by nanopore sequencing. However, despite this discrepancy, the main differences observed between the cerebellum and hippocampus in the cloning study were also detected with the nanopore method. This is shown in Fig. 5c, where the mean relative differences between these two brain structures were plotted as a ratio of the relative expression measured in each structure. Both methods (cloning vs. nanopore) reveal that some isoforms are relatively more expressed in the cerebellum (Dp71d and Dp71f), while others are more expressed in the hippocampus (Dp71d_Δ71−74_, Dp71f_Δ71_, Dp71f_Δ71−74_). An opposite result was only found for the relative expression of the Dp71d_Δ71_ isoform, which appeared overexpressed in the cerebellum according to the cloning method and conversely overexpressed in the hippocampus according to nanopore sequencing. Overall, despite these discrepancies, the two methods revealed a comparable landscape of brain transcripts and the presence of both temporal and spatial variations during brain development.

## Discussion

Dystrophin gene (*DMD*) products naturally undergo alternative splicing of specific exons, particularly between the most distal exons 68 to 79 of the genomic sequence [14, 53, 54], which leads to the existence of isoform subfamilies for each *DMD*-gene product. Fourteen isoforms have been thus identified for Dp71 [14, 15, 17, 39], the main intellectual disability-associated brain dystrophin, and at least eight of them are detected at substantial levels in the brain [17]. Although these multiple isoforms may be expressed and possibly play distinct roles in a variety of cell types and subcellular domains in the CNS [55], their function is still largely unknown. Several studies showed that the expression level of Dp71 mRNA increases during differentiation of neural PC12 cells [40, 56, 57], while antisense Dp71 RNAs may suppress PC12 neurite outgrowth [58]. Likewise, increased expression of Dp71 mRNA was also reported in differentiating astrocytes [59]. Moreover, a role for Dp71 in cell cycle and mitosis was suggested [60], and several Dp71 isoforms have been implicated in cell proliferation [40, 42, 61]. A recent study reported variable patterns of expression of the distinct Dp71 isoforms during proliferation and differentiation of neural stem cells [62], which further suggests that expression of various Dp71 isoforms may be regulated at distinct stages from cell formation to maturation.

In the present study, we report the expression of a range of Dp71 isoforms from the Dp71d (containing exon 78) and Dp71f (lacking exon 78) groups during brain development. These isoforms may also display distinct alternative splicing of exons 71 to 74 and are named according to our recently proposed nomenclature [17]: Dp71d (full-length Dp71), Dp71d_Δ71_, Dp71d_Δ74_, Dp71d_Δ71−74_, Dp71d_Δ71,74_, Dp71f (only lacking exon 78), Dp71f_Δ71_, Dp71f_Δ74_, Dp71f_Δ71,74_, and Dp71f_Δ71−74_. We show that these multiple Dp71 isoforms are expressed in the hippocampus, neocortex, and cerebellum, which are target brain structures involved in the cognitive impairments reported in DMD patients and in a mouse model lacking Dp71 [7, 26, 27, 29, 31, 36]. Previous reports showed that total Dp71 mRNA expression gradually increases from embryonic stages [5, 37, 45]. In the present study, we analyzed the relative expression of the main brain Dp71 isoforms. We thus demonstrate that their expression is regulated at specific embryonic and postnatal stages of brain development characterized by developmental landmarks of specific cellular changes, which raises new hypotheses regarding their relevance during brain development.

### Embryonic Expression of Specific Brain Dp71 Isoforms

Few seminal studies have shown that Dp71 is the first *DMD*-gene product detectable in the embryonic brain at the time of neural tube closure [5, 10, 63–65]. In one of these studies, the transgenic insertion of beta-galactosidase cassettes after the specific first exon of Dp71 in mice enabled the evaluation of Dp71 promoter activity with X-gal staining, which revealed a faint staining from E8.5 in the dorsolateral forebrain, while a prominent staining was seen at E9.5 in the forebrain and hindbrain [5]. However, these techniques used previously did not discriminate the distinct isoforms of Dp71.

In the present study, we demonstrate that the mouse embryonic brain (E10.5–E15.5) expresses Dp71d, Dp71d_Δ71_, Dp71d_Δ74_, Dp71d_Δ71−74_, Dp71f, Dp71f_Δ71_, Dp71f_Δ74_, and Dp71f_Δ71−74_. While the Dp71d group of isoforms is predominant (> 75%) in adult brain tissues [17, 55], we show here for the first time that in contrast, the Dp71f group of isoforms is predominant in the embryonic brain. Importantly, this was clearly evidenced using three types of strategies designed to quantify Dp71 splice variants, i.e., RT-PCR, cloning assays, and third-generation sequencing technologies, which excludes a potential bias due to embryonic inter-individual variability and thus strengthens this main conclusion. Moreover, we show that this predominance of the Dp71f subfamily in the embryonic brain also occurs in the rat brain, suggesting that this regulation affects the development of rodents’ brain in general.

Splicing of exon 78 changes the reading frame and results in the replacement of the last 13 hydrophilic amino acids of dystrophin with 31 new hydrophobic residues [66]. This modifies the structure of the C-terminal dystrophin tail from a β-sheet fold to an amphipathic α-helix [67]. Although this does not affect C-terminal binding of dystrophin with its main known molecular partners, this highly conserved regulation in vertebrates provides a more hydrophobic C-terminus and alters dystrophin’s capacity to maintain cell membrane integrity. Importantly, it has been reported that skipping of exon 78 may affect all dystrophins and typically occurs in a variety of early embryonic tissues [64]. In a study of myotonic dystrophy type 1 (DM1), a neuromuscular disease in which altered RNA splicing factors deregulate alternative splicing, it was shown that *DMD* exon 78 is abnormally spliced in patients’ muscles. Interestingly, this resulted in the re-expression of an embryonic Dp427 muscle dystrophin lacking the amino acid sequence coded by exon 78, in place of the adult full-length Dp427 isoform, thus compromising muscle function in this syndrome [67]. More recently, we have published a transcriptomic analysis of flow-sorted retinal photoreceptors, showing that the majority of *DMD*-gene transcripts contain exon 78 in mature photoreceptors, while immature photoreceptors preferentially express transcripts lacking exon 78, indicating that skipping of this exon preferentially occurs in immature CNS cells [55]. In all, these data suggest that isoforms with skipped exon 78, particularly those of the Dp71f group, have a critical role during development. While the absence of exon 78 may have pathological consequences in adult tissues, it might be conversely required to reduce membrane rigidity and/or associate with distinct scaffolds of membrane proteins during developmental changes in cell morphology.

In the present study, we also demonstrate that the expression of Dp71 isoforms is differentially regulated between E10.5 and E15.5. At E10.5, only two isoforms are detected: Dp71f lacking exon 78 (84%) and the full-length Dp71d to a lesser extent (16%). The Dp71f isoform, therefore, appears as the main embryonic isoform at this stage. At E15.5, however, Dp71 isoforms also include those lacking additional exons (Dp71d_Δ71_, Dp71d_Δ74_, Dp71d_Δ71−74_, Dp71f_Δ71_, Dp71f_Δ74_, and Dp71f_Δ71−74_), yet they are expressed at lower levels (< 15%) compared with Dp71f lacking only exon 78 (55%) and full-length Dp71d (45%).

E10.5 is characterized by a peak of gliogenesis that follows neural tube closure and precedes neurogenesis. At this embryonic age, polarized neuroepithelial stem cells give rise to radial glia progenitor stem cells [68]. This period also includes microglia invasion simultaneously with radial glial differentiation [69]. While Dp71 is not detected in microglia [55], its expression in radial glial cells is expected. Expression of Dp71 in developing epithelial tissues and neural stem/progenitor cells has been previously reported [62, 63, 70, 71], as well as its association with glial cells in the developing CNS [65]. Moreover, its expression in radial glial cells has been shown in both retinal Müller cells [6, 72] and cerebellar Bergmann cells [73]. Our data, therefore, suggest that Dp71f could be the main isoform expressed in brain radial glial cells at E10.5. The small quantity of Dp71d detected at this age might correspond to a restricted expression in pluripotent cells. Alternatively, Dp71d could also be present in small amounts in specific subcellular domains of radial glial cells, where it could contribute to apical/basal cell determination depending on distinct interactions of Dp71-associated protein complexes with various extracellular matrix proteins [71].

E15.5 is characterized by neural stem cells entering neurogenesis to form cortical neurons. At this age, we observed an increased presence of Dp71d (45% at E15.5 instead of 16% at E10.5). A putative role of Dp71d during neurogenesis is supported by previous studies showing the expression of this isoform in the soma and nucleus of mouse neurons in primary cultures [7, 74]. The additional detection of Dp71d/Dp71f isoforms lacking exons 71 and 71–74 at E15.5 might reflect a contribution of these isoforms to neuronal differentiation and/or proliferation processes [60–62, 75]. Alternatively, it has also been shown that an undifferentiated network of capillaries also emerges at E9–E10 and expands between E14.5 and E18.5, as the blood-brain barrier starts to develop between E11 and E17 [46, 49, 76, 77]. A putative role for Dp71 isoforms during angiogenesis is partly supported by our recent study showing the presence of Dp71 mRNA in endothelial cells [55]. Although this previous study was undertaken using cortical cells purified at postnatal day 17, embryonic expression of Dp71 in endothelial domains has been reported in dogs, at an age equivalent to mouse days 14–16 [71].

In all, we show that the Dp71f isoform predominates at E10.5 and is then progressively downregulated from embryonic to adult brain, while the Dp71d isoform is upregulated from E15.5, and the Dp71d group finally reaches a relative expression of 76% in the adult brain. Hence, the Dp71f isoform likely has a key role at E10.5, when radial glial cells and a primitive capillary network develop, while the first important upregulation of Dp71d coincides with the peak of neurogenesis at E15.5, suggesting a role for this full-length Dp71 isoform in the proliferation of neuronal cells.

### Expression of Distinct Brain Dp71 Isoforms Is Regulated During the Postnatal Period

The postnatal period also witnessed differential modification in the expression levels of several Dp71 isoforms, which further strengthens the hypothesis that splicing events affecting Dp71 mRNAs have relevance for brain development. We found that the Dp71d group predominates throughout the postnatal period. However, substantial variations of the Dp71d/Dp71f ratio were observed across ages and between the three brain structures analyzed here (cortex, hippocampus, and cerebellum). The multiple cellular and network modifications that occur during the postnatal period, as depicted in the top diagrams of Figs. 3 and 4, make interpretations of the results more challenging, as Dp71 isoforms may be differentially expressed in glial, vascular, and synaptic elements [7, 78]. Moreover, the differences observed between cortical, subcortical, and cerebellar structures may potentially relate to the main differences in the dynamics of these developmental processes in these distinct structures.

The relative expression of the two main groups of Dp71 isoforms, Dp71d and Dp71f, showed substantial variations across postnatal ages. At P7, we detected a slight increase in the expression of the Dp71d group in the hippocampus, while an increased expression of the Dp71f group was observed in the cortex and cerebellum. Interestingly, AQP4 is detected during development starting at E16, which may correspond to the time at which the Dp71d isoform shows a marked increase in its expression (quantified at E15.5 in our study), and GFAP-positive perivascular astrocytes display highly polarized expression of AQP4 at P7 [46, 79, 80]. However, it remains uncertain as to whether changes in expression levels of these Dp71 isoforms reflect the maturation of perivascular astrocyte endfeet or an involvement during postnatal remodeling of brain vasculature and/or synaptogenesis [47, 77, 81, 82]. At P21, we found a marked increase in the relative expression of the Dp71f group of isoforms, but selectively in the cerebellum. P21 is globally associated with a decay of synaptogenesis in cortical and subcortical structures. In the cerebellum, however, some processes, such as the formation of the Purkinje-cell monolayer [83] and the remodeling of climbing and parallel fiber synaptic connections onto Purkinje cells [84], are not fully achieved. This suggests a putative role for Dp71f isoforms in these cerebellar synaptic processes. The expression of Dp71f in synaptic domains is supported by a study showing its selective expression in postsynaptic elements in neuronal cultures [7].

Our approach also allowed us to detect the regulated expression of specific isoforms within the Dp71d and Dp71f groups during the postnatal period. In addition to the full-length Dp71d and exon78-lacking Dp71f isoforms, both groups included two main additional isoforms spliced out for exon 71 (Δ71) or exon 71 to 74 (Δ71–74). We previously have shown the presence of these spliced-out transcripts and the relatively large expression of Dp71d_Δ71−74_ (also called Dp71c) in the adult brain [17]. Here, we noticed differences in their relative levels of expression between brain structures. The causes of these differences remain to be studied, but one may hypothesize that they are related to the presence of distinct Dp71 isoforms in specific subpopulations of cells having variable densities in distinct regions. We selected samples from adult hippocampus and cerebellum to compare cloning assays and third-generation sequencing technologies in their potential to detect this type of variations, since we already found both methods efficient to quantify differences between embryonic and adult brains for the two groups of Dp71 isoforms. Here, the two methods identified the same isoforms, but provided different raw expression levels, suggesting differences in mRNA abundance estimation. Obviously, the relative expression levels of the six individual Dp71 isoforms were lower than those quantified when isoforms were collectively analyzed as groups. However, the largest differences observed between the two brain structures were confirmed by the two methods, showing higher levels of expression of Dp71d and Dp71f in the cerebellum and a larger expression of Dp71d_Δ71−74_ in the hippocampus compared with the cortex and cerebellum (this larger expression of Dp71 isoforms lacking exons 71–74 in the hippocampus was also confirmed by RT-PCR in adult tissues). Both the cloning and nanopore methods, therefore, have the potential to estimate variations between developmental ages and between brain structures.

Importantly, Dp71d, Dp71f, and the isoforms lacking exon 71 or 71–74 have also been characterized at the protein level in other studies [7, 15, 17, 61, 66, 78, 85]. The lack or presence of exon 71 or 71–74 likely has important functional consequences. Indeed, these exons encode the C-terminal domain responsible for Dp71 binding to the signal-transducing adaptor protein, syntrophin, involved in clustering membrane channels and receptors in a variety of cell types [86].

In the hippocampus, Dp71d and Dp71d_Δ71_ showed a large expression from P1 to P14 (20–40%), suggesting a relevance during periods of astrocytogenesis, angiogenesis, and/or dendritic growth, as well as during the formation of the dentate gyrus layers [87]. It is worth noting that even if astrocytogenesis mostly occurs between E16 and P7, the polarized expression of AQP4 channels in perivascular astrocytes is only completed at P14 and likely involves interactions with isoforms of the Dp71d subfamily [33]. Maturation of astrocyte gap junctions may continue until P19 according to the expression of specific connexins, and this requires interactions with dystrophin-associated proteins including the Dp71-associated syntrophins [88, 89]. In contrast, Dp71d_Δ71−74_ expression particularly peaked at P21 (71%), a period of intense spinogenesis, and remained high in the adult hippocampus (49%). Likewise, expression of the Dp71f_Δ71−74_ also peaked at P21, yet it remained at a low level (14%) compared to Dp71d_Δ71−74_. Knowing that the hippocampus is characterized by intense synaptic plasticity associated with learning processes, these results suggest that this isoform that misses the capacity to bind syntrophins is required during synapse remodeling. One hypothesis that could be considered in future studies is the involvement of these isoforms in a molecular switch system, allowing more membrane fluidity for the trafficking of membrane proteins during synapse structural and functional plasticity, while full-length isoforms with syntrophin-binding capacity would favor protein stabilization at the postsynaptic density of dendritic spines. For example, Dp71 isoforms lacking exon 71 are localized in the cell membrane and cytoplasm and colocalized with β-dystroglycan and α1-syntrophin in PC12 cells, while Dp71 isoforms lacking exons 71 to 74 are exclusively localized in the cell membrane and do not colocalize with β-dystroglycan and α1-syntrophin [39].

Different patterns of variations were observed in the cortex and cerebellum. In the cortex, Dp71d expression peaked at P60 and that of Dp71d_Δ71_ at P14 and P60. Dp71d_Δ71−74_ showed a larger expression between P1 and P21 (34–53%) as compared to adults (22%), while Dp71f_Δ71−74_ peaked at P7 (25%). In the cerebellum, Dp71d peaked at P14 and P60, Dp71d_Δ71−74_ at P1–P7, and Dp71f_Δ71−74_ at P7 and P21. As stated above, these regional differences may be attributed to a selective expression of distinct Dp71 isoforms in separate cell types that may be differentially enriched in distinct brain structures, or because of the variable dynamics of developmental processes in distinct brain structures. Although the final volumes of the hippocampus, cortex, and cerebellum are achieved by P21 [90], late neuronal cell maturation is observed particularly in the cerebellum [82, 91, 92]. Nevertheless, the results confirm that specific Dp71 isoforms are differentially regulated across postnatal ages, including during adulthood when activity-dependent plasticity events may occur within neuronal networks.

## Concluding Remarks

This study demonstrates that the exon 78 spliced-out, in Dp71f isoforms, has a major expression in the embryonic rodent brain. Moreover, we show that six main Dp71 isoforms have a differentially regulated expression from E15.5 to postnatal and adult ages in the mouse brain. Our findings, therefore, support the current hypothesis that distinct isoforms may play complementary roles in several cell types, as well as during specific steps of cell proliferation, differentiation, and structural plasticity. Because various Dp71 isoforms may be expressed in radial glia, astrocytes, endothelial cells, oligodendrocytes, and neuronal synapses [55], the specific roles of each isoform remain hypothetical, considering the multiple cellular events taking place in parallel during brain development. However, our findings highlight that it will be worth in future studies to decipher the respective expression of Dp71 isoforms in distinct cell types during brain development, including that of the Dp40 isoform that was not analyzed in the present study. Putative overlaps with time-dependent changes in the expression of syntrophin and AQP4 should also be considered, to further specify the functional link between Dp71 isoforms’ regulation and the maturation of the blood-brain barrier.

Future studies could benefit from recent techniques with improved accuracy, such as third-generation (long-read) sequencing that has the potential to identify full-length and alternatively spliced transcripts, and for which we show here that it holds promising prospects for rapid evaluation of quantitative regulations of Dp71 isoforms during brain development. These technologies have recently undergone active development and, coupled with spatial single-cell transcriptomics, will likely resolve the issue of understanding the different regulations observed in distinct brain structures and cell types [93]. Modulating the expression of specific isoforms using RNA interference in wild-type animals and ectopic expression in Dp71-deficient mice, or developing new transgenic models missing selective isoforms, might also be considered complementary approaches to apprehend the multifarious functions of alternatively spliced Dp71 isoforms.

Activity-dependent splicing emerges as a possible posttranscriptional process enabling rapid regulation of the levels and dynamics of specific gene products, to modulate the cell transcriptional repertoire at specific developmental stages and the cell’s connectivity and functions [94, 95]. In this regard, the multiple splicing events affecting *DMD*-gene transcripts may represent an attractive model to understand how alternative splicing impacts protein function and thus affects cell functional properties. Our present study supports our previous in vitro findings suggesting that Dp71 splicing events may be part of remodeling processes required for cell proliferation, differentiation, adhesion, and neurite formation [8]. Our previous studies using cell cultures also unveiled that distinct Dp71 isoforms may have different subcellular localization and colocalization with components of the dystrophin-associated protein complex, such as with dystroglycan and syntrophin proteins, depending on their specific C-terminal end and to the occurrence of exons 71–74 splicing [7, 39, 70]. Obviously, our understanding of the functional relevance of Dp71 posttranscriptional modifications is still incomplete. Nevertheless, there is now converging data suggesting that distinct Dp71 isoforms may participate in the regulation of cellular mechanisms involved in developmental processes, which warrants future investigations of this subfamily of dystrophins associated with central comorbidities in DMD.

## Electronic Supplementary Material

Below is the link to the electronic supplementary material.

Supplementary Material 1

## Data Availability

Datasets available on request to the authors.
