# Supplementary material for: Expression of Dystrophin Dp71 Splice Variants Is Temporally Regulated During Rodent Brain Development
Source: Mol Neurobiol. 2024 May 28;61(12):10883–900. doi: 10.1007/s12035-024-04232-2 (PMC11584426; doi:10.1007/s12035-024-04232-2)
Supplement: Supplementary file 1 — Supplementary Material 1 [file 12035_2024_4232_MOESM1_ESM.pdf]

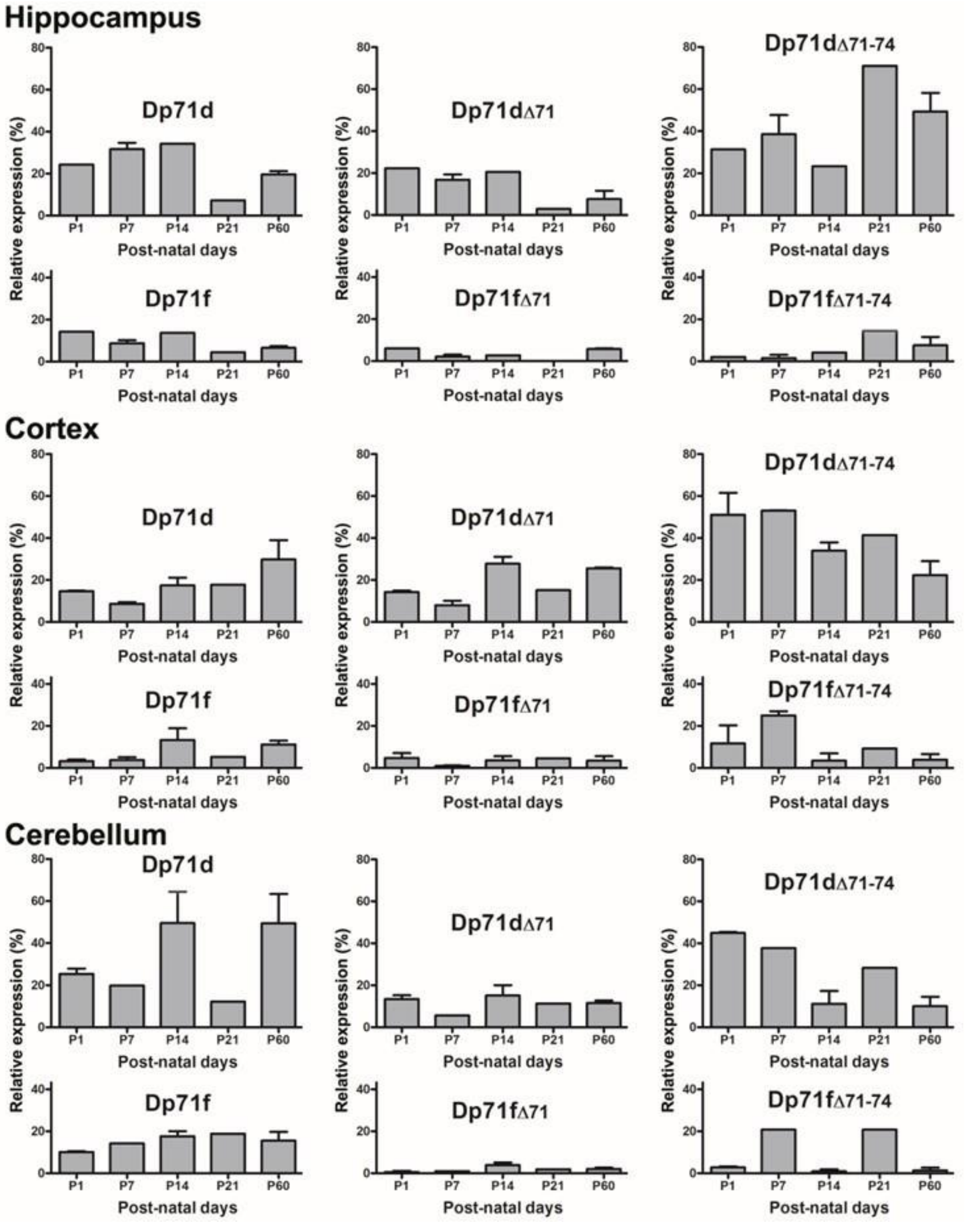

Supplementary Figure S1 Relative expression of Dp71 isoforms. Graphs show the relative expression (%) of Dp71d and Dp71f isoforms most frequently expressed during the postnatal development of mouse hippocampus, cortex and cerebellum. Data are shown as percent or mean percent of the relative frequency plus SD

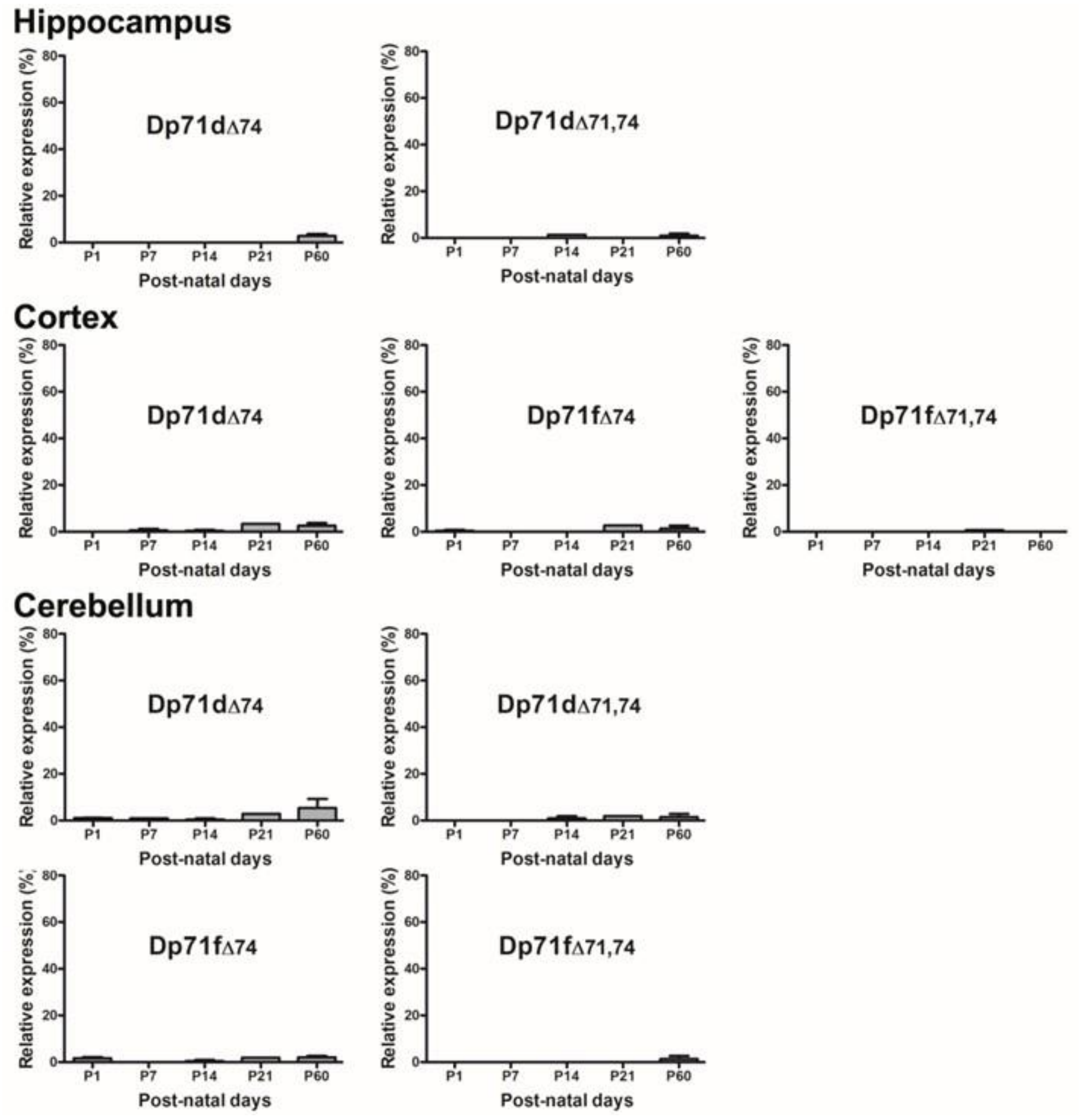

Supplementary Figure S2 Relative expression of Dp71 isoforms. Graphs show the relative expression (%) of Dp71d and Dp71f isoforms with a low expression during the postnatal development of mouse hippocampus, cortex and cerebellum. Data are shown as percent or mean percent of the relative frequency plus SD

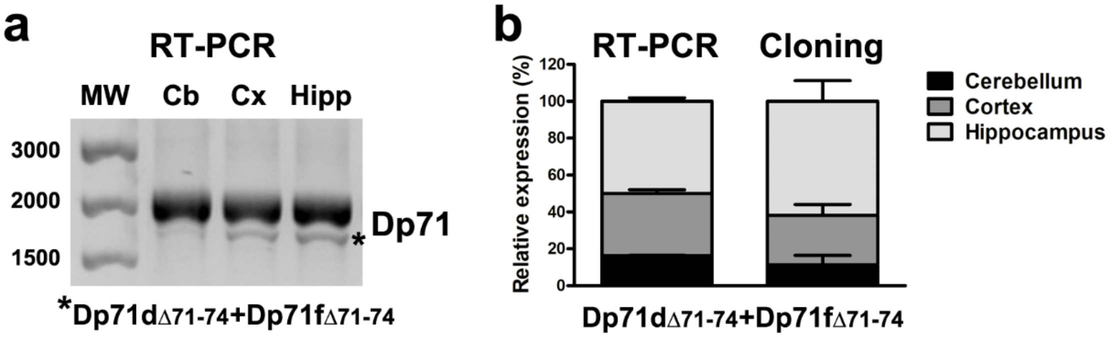

Supplementary Figure S3. Relative expression of all Dp71 isoforms spliced out for exons 71 to 74 (Δ71-74) in adult mouse (P60) brain tissues. **a** Expression of Dp71d$_{\Delta 71\text{-}74}$ and Dp71f$_{\Delta 71\text{-}74}$ isoforms (*lower band) were collectively detected by RT-PCR as a band with lower intensity compared to full-length Dp71 (upper band). Cb: cerebellum, Cx: cortex and Hipp: hippocampus. **b** Relative expression (%) of Dp71d$_{\Delta 71\text{-}74}$ plus Dp71f$_{\Delta 71\text{-}74}$ isoforms detected by RT-PCR and cloning experiment in cerebellum, cortex and hippocampus. Densitometric scanning was obtained using ImageJ. Data are shown as mean percent plus SD
